# Monochromatic “Photoinitibitor”‐Mediated Holographic Photopolymer Electrolytes for Lithium‐Ion Batteries

**DOI:** 10.1002/advs.201900205

**Published:** 2019-04-04

**Authors:** Ronghua Yu, Sibo Li, Guannan Chen, Cai Zuo, Binghua Zhou, Mingli Ni, Haiyan Peng, Xiaolin Xie, Zhigang Xue

**Affiliations:** ^1^ Key Laboratory for Material Chemistry of Energy Conversion and Storage Ministry of Education School of Chemistry and Chemical Engineering Huazhong University of Science and Technology Wuhan 430074 China; ^2^ School of Materials Science and Engineering Wuhan Institute of Technology Wuhan 430074 China

**Keywords:** holography, ordered structure, photoinitibitor, polymer electrolyte

## Abstract

A new polymer electrolyte based on holographic photopolymer is designed and fabricated. Ethylene carbonate (EC) and propylene carbonate (PC) are introduced as the photoinert substances. Upon laser‐interference‐pattern illumination, photopolymerization occurs within the constructive regions which subsequently results in a phase separation between the photogenerated polymer and unreacted EC–PC, affording holographic photopolymer electrolytes (HPEs) with a pitch of ≈740 nm. Interestingly, both diffraction efficiency and ionic conductivity increase with an augmentation of the EC–PC content. With 50 wt% of EC–PC, the diffraction efficiency and ionic conductivity are ≈60% and 2.13 × 10^−4^ S cm^−1^ at 30 °C, respectively, which are 60 times and 5 orders of magnitude larger than the electrolyte without EC–PC. Notably, the HPEs afford better anisotropy and more stable electrochemical properties when incorporating *N*,*N*‐dimethylacrylamide. The HPEs exhibit good toughness under bending, excellent optical transparency under ambient conditions, and astonishing capabilities of reconstructing colored images. The HPEs here open a door to design flexible and transparent electronics with good mechanical, electrical, and optical functions.

## 1 Introduction

Nowadays, lithium‐ion batteries (LIBs) are the first choice as the power supply for portable electronic devices. They dominate the entire electronics market due to their high energy density, stable cycling performance, relatively environmental friendliness, etc.^1^ Compared with liquid electrolytes, polymer electrolytes are more promising for future LIBs because they are able to offer outstanding advantages such as better safety, excellent stability, and facile processability.^2^

Poly(ethylene oxide) (PEO) was first studied in polymer electrolytes by Wright and co‐workers in 1973.^3^ The complexation between PEO and alkali metal salts could facilitate the ionic conductivity. Since then, a myriad of efforts have been devoted to the development of new polymer electrolytes based on PEO, by considering the ion transfer mechanism and electrical properties.^4^ To improve the ionic conductivity, many strategies have been developed to suppress polymer crystallization. For instance, polymer blends, block copolymers, comb‐like polymers, and cross‐linked network polymers are designed toward the target.^5^ In addition, strategies such as replacing polymer matrices, employing new lithium salts, and loading plasticizers have been considered.^6^ In particular, gel polymer electrolytes formed by adding plasticizers or solvents to the polymer matrix have attracted great interests because of their higher ionic conductivity.^7^ However, the increase in ionic conductivity is at the expense of mechanical strength, which contradicts the strategy of using polymer electrolytes with good mechanical properties for safer LIB applications. Therefore, alternative strategies are required to balance the ion transport and mechanical strength of polymer electrolytes.

The construction of ion channels in polymer electrolytes provides a new concept for the design of new polymer LIBs. The formation of channels promotes the ion conduction in the electrolyte and thus reduces the barrier effect. Balsara and co‐workers used the energy‐filtered transmission electron microscopy to study the Li^+^ distribution in polystyrene‐*b*‐PEO (PS‐*b*‐PEO) block copolymer electrolytes with the layered morphology.^8^ They have demonstrated that ion conducting channels can be formed in the PEO domain, utilizing the PS‐*b*‐PEO copolymer microphase separation at the 10–100 nm scale. Owing to the combination of ion transport channels and a high modulus of PS segments, the high ionic conductivity and good mechanical properties are achieved. Supramolecular self‐assembly is also a good way to prepare nanochannel in polymer materials. Chen and co‐workers developed a crystalline polymer electrolyte through the supramolecular self‐assembly of PEO, α‐cyclodextrin (α‐CD), and LiAsF_6_.^9^ The nanochannels formed by α‐CD for the directional motion of Li^+^ ions further promote Li^+^ transport, and at the same time prevent the transport of large anions due to the relatively small cavity size of the channel. Besides, PEO‐based electrolytes combined with mesoporous silica nanoparticles possess nanoporous channels that maintain a high surface area and pore volume, which allow for facile transportation of lithium ions.^10^ Although these technologies provide elegant solutions for ion channel construction, there are still some intrinsic shortcomings, including, e.g., poor long‐range order, lack of channel size regulation, and complex phase structures.

Li and co‐workers proposed polymer electrolytes with long‐range ordered ion channels based on holographic photopolymerization–induced phase separation.^11^ However, phase segregation is dominantly influenced by diffusion during holographic recording. The increased viscosity by the macromolecular PEO which was used as inert parts during holographic recording would decrease the diffusion rate, and the phase separation is thus far from that designed. Clearly, for future battery applications, new simple paradigms are highly awaited that are able to afford good mechanical, electrical, and optical functions to a polymer electrolyte. Laser holography has been widely used in technical fields such as image storage, security, and micro‐nanomanufacturing.^12^ Recently, we proposed a novel, visible light sensitive “photoinitibitor,” which is able to simultaneously generate a radical with the initiation function and another radical with the inhibition function.^13^ During holographic photopolymerization, it is critical to implement a precise spatiotemporal control over the polymerization kinetics and gelation. What is the most important in this regard is that the gelation time difference between the constructive and destructive regions should be amplified. A relatively fast gelation in the constructive regions enables fast holographic reconstruction, while the prolonged gelation in the destructive regions boosts the diffusion and phase separation. Quite recently, we have disclosed that several photoinitiating systems hold the unique simultaneous photoinitiation and photoinhibition functions.^14^ Nevertheless, their advantages to regulate the polymer electrolytes have not been realized.

**Figure 1:**
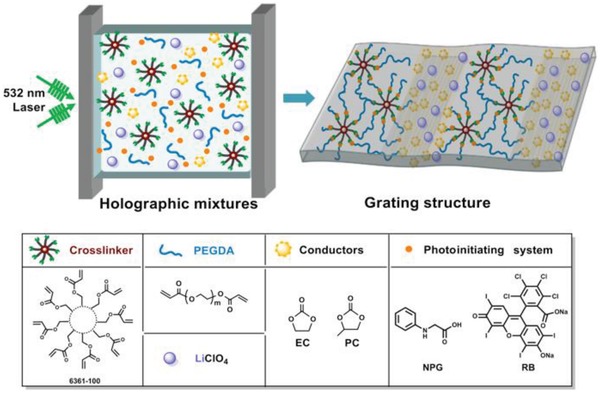
Holographic photopolymerization strategy for fabricating polymer electrolytes under green light.

Herein, we introduce a small molecular mixture of ethylene carbonate (EC) and propylene carbonate (PC) into the holographic photopolymer system to produce a holographic photopolymer electrolytes (HPEs) with long‐range ordered ion channels (**Figure** **1**). The PEO segment was cross‐linked by the polymer network to offer good mechanical properties. To obtain the HPEs with a high degree of phase separation, the rose bengal (RB)/*N*‐phenylglycine (NPG) system with simultaneous initiation and inhibition functions was employed to mediate the laser holographic recording.^14^ The concurrent photoinitiation and photoinhibition functions upon a monochromic light irradiation have been demonstrated to be critical in exerting a precise control over the photopolymerization kinetics, gelation, and phase separation during holographic recording.^13^ During the holographic polymerization process, holographic mixtures comprising EC–PC and PEO‐based reactive oligomer are exposed to two coherent green laser beams (532 nm). Mixed monomer and reactive oligomers continuously migrate to the constructive (bright) regions to participate in the photopolymerization while squeezing the EC–PC into the destructive (dark) regions from the constructive regions. Due to the addition of EC–PC as the inert material, a polymer material with periodic structures is formed. First, in such a periodic structure, EC–PC is regarded as an ion channel, enabling polymer materials to be used in LIBs. Second, the refractive index difference is formed between the EC–PC and the polymer collective, imparting holographic optical properties such as diffraction characteristic to the photopolymer electrolyte. The high diffraction efficiency of the photopolymer electrolyte allows for the storage of colored images that are easily recognizable by the naked eye under white light. Very interestingly, the more distinct grating structure and improved specific capacity LIBs were obtained when incorporating a polar monomer (such as *N*,*N*‐dimethylacrylamide, DMAA) into the holographic system. The paradigm presented in this work paves a way for the design of polymer electrolytes with long‐range ordered ion channels, and demonstrates the multifunction of polymer materials through holography.

## 2 Results and Discussion

### 2.1 Appearance of Holographic Photopolymer Electrolytes

**Figure 2:**
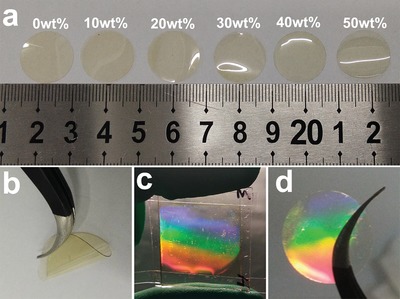
a) Photographs of HPEs with varied content of EC–PC, b) bending of HPEs with 30 wt% of EC–PC, c,d) rainbow colors diffracted by the HPEs with 30 wt% of EC–PC under white light.

A visible light sensitive “photoinitibitor” (e.g., RB/NPG) simultaneously generates two distinct radicals upon monochromatic green light exposure. The photoinitiation and photoinhibition processes are critical to control polymerization kinetics, gelation, and phase separation during holographic recording. Six HPE membranes with varied contents of EC–PC from zero to 50 wt% were prepared (**Figure** **2**a and Tables S1–S4 (Supporting Information)). They show a good transparency under ambient conditions and exhibit attractive toughness when bending (Figure 2b). In addition, the mechanical property was also used to evaluate the performance of HPE. Compared with PEO‐based solid polymer electrolyte which was obtained through the radical cross‐linking polymerization of poly(ethylene glycol) diacrylate_400_ (PEGDA_400_), HPE shows greater elongation at break even with 50 wt% of small molecular EC–PC (Figure S1, Supporting Information). The tensile stress of HPE sample reaches to 6.5 MPa when EC–PC was removed. These astonishing characteristics promise their applications in transparent and flexible electronics. Interestingly, they display rainbow colors under white light illuminations due to the light diffraction from the gratings inside the photopolymer electrolyte (Figure 2c,d). This phenomenon indicates a microphase separation between the polymer and inert parts (EC–PC and/or lithium perchlorate (LiClO_4_)). In addition, it is exciting that the introduction of polar monomer has little effect on the appearance of HPEs. To be noted, rainbow colors arise here because of the varied incident angle of the white light for each part of the large area holographic photopolymer electrolyte films, rather than the varied grating pitch reported in other materials.^15^

### 2.2 Holographic Performance

**Figure 3:**
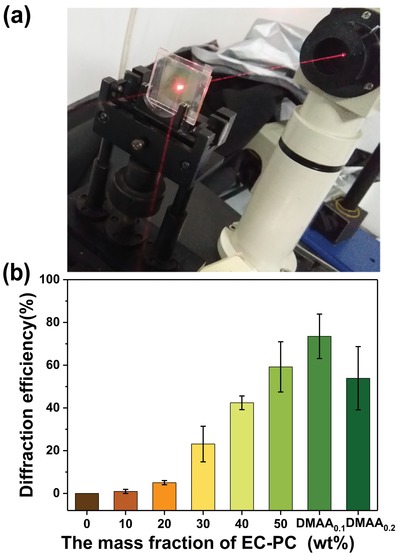
a) Real setup to characterize the diffraction efficiency, b) diffraction efficiency of holographic photopolymer electrolytes with varied content of EC–PC. The proportion of 50 wt% of EC–PC in DMAA_0.1_ and DMAA_0.2_ is the same.

When a beam of 633 nm He–Ne laser illuminates the HPEs, both the transmitted and diffracted beams are clear (**Figure** **3**a). The diffraction intensity can be changed by rotating the sample stage. When the diffraction intensity reaches its maximum during the stage rotation, the angle between the incident beam and the normal of the sample plane is referred as the Bragg angle. Then, the diffraction efficiency is calculated to be the ratio of diffraction intensity to the total intensities of diffraction and transmission at the Bragg angle. The EC–PC is found to be critical to offer a high diffraction efficiency to the photopolymer electrolyte. As displayed in Figure 3b and Table S5 (Supporting Information), no detectable diffraction is given in the photopolymer electrolyte without EC–PC. When increasing the EC–PC content from 10 to 50 wt%, the diffraction efficiency is detected to be increased from 0.9 ± 0.9% to 59.2 ± 11.7%. The diffraction efficiency increases around 60 times. The refractive index modulation also increases 8 times (i.e., from 0.00018 to 0.00167). Notably, the diffraction efficiency (73.5 ± 10.4%) in the holographic photopolymer electrolyte when incorporating DMAA (HPE–DMAA) is much higher than that of HPE without DMAA. As displayed in Figure S2 (Supporting Information), the photopolymerization is promoted due to the incorporation of small size DMAA. However, the cross‐linking reaction would be hindered with increasing the DMAA content, resulting in lowered diffraction efficiency (53.9 ± 14.8%). These results indicate that the phase separation primarily occurs between the photogenerated polymer and the unreacted EC–PC. Interestingly, there is no significant variation on the Bragg angle for each grating, indicating a similar volumetric shrinkage during photopolymerization. In principle, increasing the EC–PC content is beneficial to increasing the refractive index difference between the constructive (bright) and destructive (dark) regions of the interference pattern, thus affording a high refractive index modulation and a high diffraction efficiency to the holographic photopolymer electrolyte.

### 2.3 Micromorphology of Holographic Photopolymer Electrolyte

**Figure 4:**
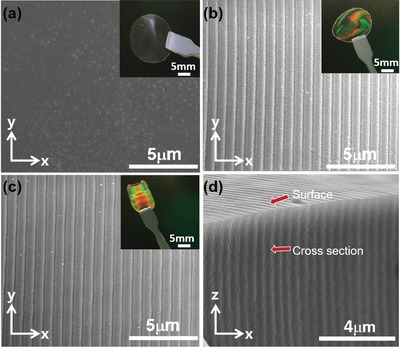
Micromorphology images of the HPE: a) SEM image of isotropic electrolytes without EC–PC, b) SEM image of anisotropic electrolytes with 50 wt% of EC–PC, c) SEM image of anisotropic electrolytes with 50 wt% of EC–PC and 0.1 g of DMAA, and d) cross‐sectional SEM image of anisotropic electrolytes with 50 wt% of EC–PC and 0.1 g of DMAA.

To confirm the structure, the scanning electron microscopy (SEM) and atomic force microscopy (AFM) were performed on the *xy*‐plane (parallel to the film surface) and the *xz*‐plane (vertical to the film surface) of the films (see Figure S3 in the Supporting Information). As proof of the key role of EC–PC, we prepared two types of photopolymer electrolytes, namely, one without EC–PC and another with 50 wt% of EC–PC. **Figure** **4**a–c shows SEM images of *xy*‐plane of the film. No grating structures can be observed in the absence of EC–PC due to the absence of photoinert substances. In contrast, the uniform grating structure is clear in the holographic photopolymer electrolyte (Figure 4b,c). SEM image of the *xz* cross‐section of HPE–DMAA film is shown in Figure 4d, indicating that the direction of the aligned ionic channels is vertical with the HPE film. EC–PC is chosen here on the basis of two main reasons. First, the EC–PC is employed as an inert part to tune a phase separation between the photogenerated polymer and unreacted EC–PC. This approach enhances a microphase separation between the polymer and EC–PC. Second, EC and PC have high relative permittivities, and can also enhance the dissociation of ion pairs of salts. EC or PC is thus widely used as a plasticizer in polymer‐based electrolytes for LIBs. The periodic spacing is determined to be ≈700 nm (Figure S4a, Supporting Information). However, the channel in Figure S4b (Supporting Information) is smoother owing to the higher diffraction efficiency of HPE–DMAA, which is beneficial to conducting lithium ions. The darker channels in Figure 4 are the left area after removing the EC–PC by acetonitrile for 72 h. The AFM was also employed to identify the morphology (Figure S5, Supporting Information). The grating spacing is determined to be ≈740 nm, in good agreement with the SEM characterization.

### 2.4 Ionic Conductivity

**Figure 5:**
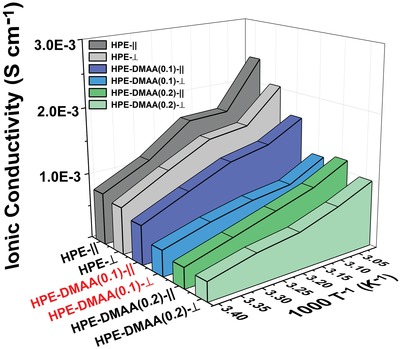
Anisotropic conductivity of HPE with varied contents of DMAA.

The anisotropic ionic conductivity of HPE was investigated by varying the ratio of EC to PC (Figures S6 and S7, Supporting Information). The anisotropic conductivity of the HPE was measured by the electrochemical impedance spectroscopy (EIS), in which copper sheets were used as blocking electrodes. The bulk resistance (*R*_b_) of HPEs can be read directly from the measured EIS. The anisotropic conductivity is significantly increased when the polar monomer (DMAA) was incorporated into the photopolymerization system (**Figure** **5**). The employment of DMAA was critical to dissolve RB and NPG completely. The improved anisotropic conductivity in the DMAA case results from the increase in diffraction efficiency and the smooth channel.

**Figure 6:**
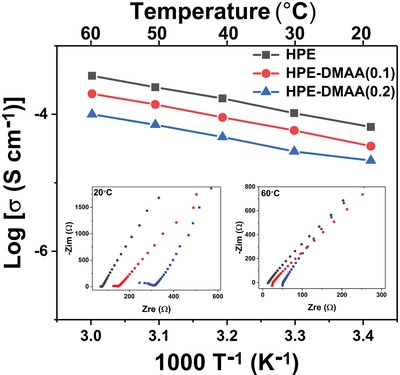
Temperature dependence of ionic conductivity for HPEs with various mass fractions of DMAA.

The ionic conductivity is a crucial parameter for the application of polymer electrolytes in LIBs. Interestingly, a very low ionic conductivity of 2.01 × 10^−9^ S cm^−1^ at 30 °C is obtained in the photopolymer electrolyte without any EC–PC (Table S2, Supporting Information). In contrast, once increasing the EC–PC content to 50 wt%, the ionic conductivity increases 5 orders of magnitude to 2.13 × 10^−4^ S cm^−1^. For the photopolymer electrolytes at 80 °C, more than 3 orders of magnitude increase in the ionic conductivity is noted when increasing the EC–PC content to 50 wt%. Figure S8a (Supporting Information) presents the temperature dependence of ionic conductivities for HPEs with varied contents of EC–PC. Notably, ionic conductivity of the HPE linearly increased with the temperature in the studied temperature range, indicating that the temperature dependence of ionic conductivities of the HPEs follows the Arrhenius equation. Figure S8b (Supporting Information) shows the ion transfer activation energy of the holographic photopolymer electrolytes as a function of EC–PC content. It is observed that as the EC–PC content increases, the energy required for lithium‐ion transfer continues to decrease. When the EC–PC content reaches 50 wt%, the activation energy is the lowest value of 27.48 kJ mol^−1^, in our photopolymer electrolytes. These results indicate that more EC–PC facilitates the ion transport. As shown in **Figure** **6**, as the amount of DMAA increases, the electrical conductivity of the HPE–DMAA decreases, which is consistent with the conclusions of the Figure 5. The lithium ion is hard to move in the polymer segments of HPE–DMAA with the higher glass transition temperature (Figure S9, Supporting Information).^16^ Figure S10a (Supporting Information) shows the proposed Li^+^ cation transport mechanism in the HPE and HPE–DMAA. The Li^+^ is conducted through the movement of ethoxylated segments and EC–PC. However, the ability of the ethoxylated segments to bind lithium cations was impaired, resulting from the addition of the DMAA. The new complexes will be formed, as shown in Figure S10b,c (Supporting Information). The competition between the ethoxylated segments and the acrylamide chains combined with lithium cation is not beneficial to raising the ionic conductivity.^17^

### 2.5 Cell Performance of Holographic Polymer Electrolyte

**Figure 7:**
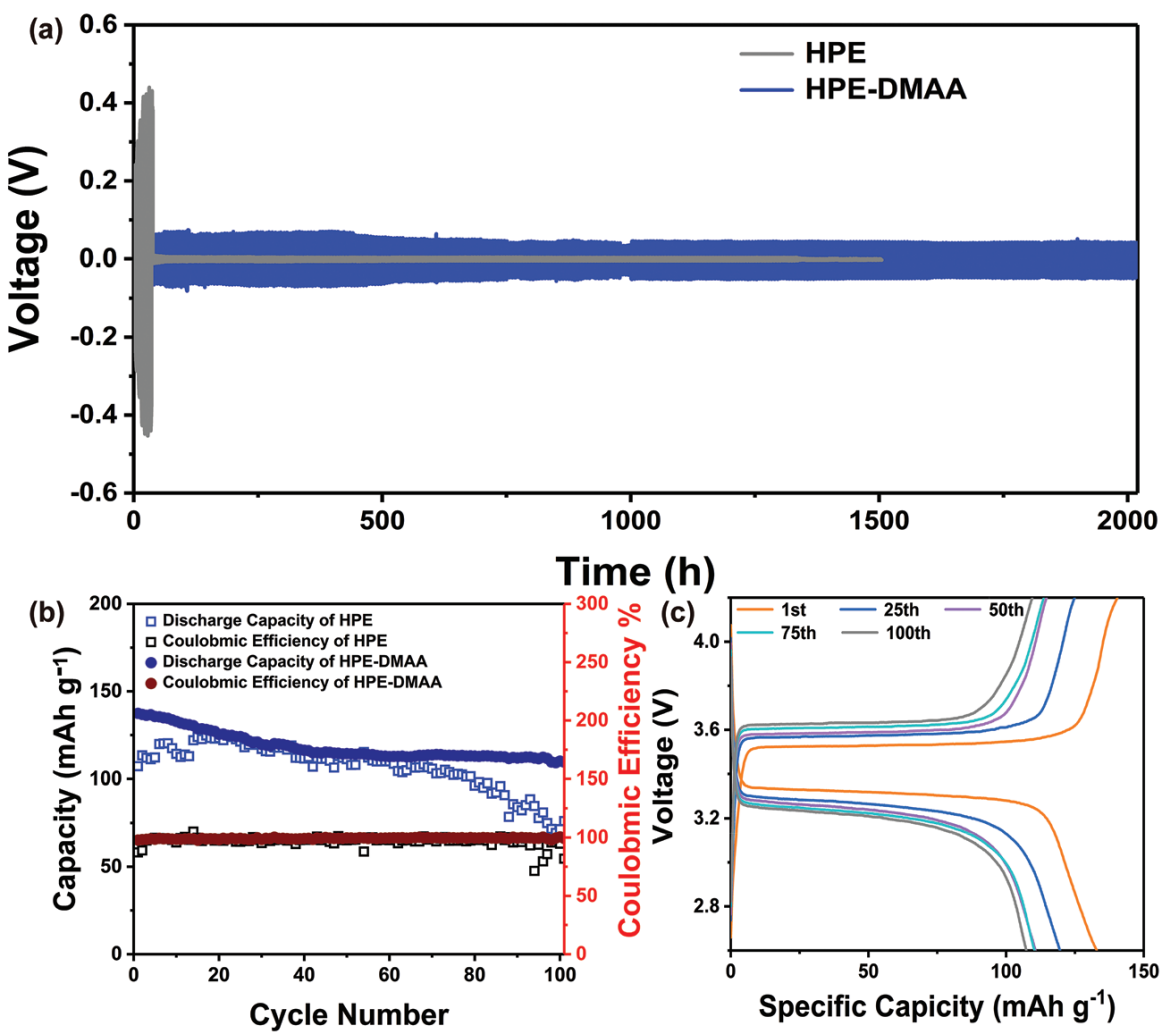
Cell performance of HPE–DMAA and HPE: a) chronopotentiometry result of Li/HPE–DMAA/Li and Li/HPE/Li symmetrical cells at the current density of 0.05 mA cm^−2^; b) the LiFePO_4_/Li cells assembled from the HPE–DMAA and HPE cycle performance during galvanostatic cycling at 0.1 C rate; c) charge and discharge profiles of HPE–DMAA at 0.1 C rate. All the measurements were conducted at 60 °C.

It is generally known that the inhomogeneous Li‐ion transport results in the overgrown Li dendrites and homogeneous Li‐ion transport can account for a uniform Li nucleation and a nondendrite growth.^18^ Therefore, understanding the lithium's stripping and plating process of HPE‐based batteries is significant. **Figure** **7**a shows the cycling performance of Li/HPE–DMAA/Li and Li/HPE/Li symmetric cells at a current density of 0.05 mA cm^−2^ under 60 °C. The cycling was prolonged to at least 2000 h without serious polarization, and no short circuit occurs for Li/HPE–DMAA/Li cell. The voltage decreased from 70 to 50 mV after 800 h. On the contrary, the voltage increased from 300 to 400 mV and short circuit occurred after 32 h for Li/HPE/Li cells. This phenomenon may be due to that DMAA can forms effective solid electrolyte interface (SEI) films, thus inhibiting further decomposition reactions of the electrolyte.^19^ Moreover, the Li‐metal surface shows an overall morphology with the ordered dendrite distribution, which is consistent with the ordered ion channel (Figure S11, Supporting Information). But compared to Li/HPE/Li cell, dendrites are not mortal for practical applications of Li/HPE–DMAA/Li cell. There are two possible explanations for the slight dendrites inside the HPE–DMAA/Li symmetric cell: 1) The flux of Li ion in photopolymer results in the inhomogeneous Li‐ion transport, thus forming Li dendrites. 2) Although the width of the formed ion channel is only ≈740 nm, the length region is still large. Therefore, the inhomogeneous Li‐ion transport still exists in the direction parallel to the ion channel. To confirm the practicability of the holographic photopolymer electrolytes, the cell performance is further evaluated in a solid‐state battery using the synthesized holographic photopolymer electrolytes. The electrochemical stability was evaluated using linear sweep voltammograms (LSVs) at 60 °C. It shows that the electrochemical decomposition potential is about 5.0 V (Figure S12, Supporting Information), indicating that the HPE has high electrochemical stability and great endurance to high voltage. The electrolytes work as separators in the CR2032 coin cell. Lithium iron phosphate (LiFePO_4_) works as the cathode, while the metal Li works as the counter and reference electrodes. The cells were cycled over a voltage range of 2.5–4.2 V at 60 °C. As shown in Figure 7b, the HPE–DMAA‐based cell displayed good cycling performance, and the initial discharge capacity was about 137 mAh g^−1^ and the capacity was still kept as high as 110 mA hg^−1^ after 60 cycles (Figure 7b,c). This reduction in capacity may be the responsibility of the evolution of dead Li from dendrites. However, HPE‐based cell only showed an initial discharge capacity of ≈120 mA h g^−1^, and it faded quickly after 60 cycles. The results manifest that the HPE–DMAA‐based cell provided a higher initial discharge capacity and better cycling stability than the HPE‐based cell, which is consistent with the conclusion of lithium deposition.

Besides, the holographic photopolymer electrolyte is ready to reconstruct color images. As displayed in Figure S13 (Supporting Information), the storage of four images is successfully achieved on the holographic photopolymer electrolytes. It shows that EC–PC can be employed as functional additives for designed holographic photopolymers with high storage capability outside of the battery application where polymer electrolyte with the image storage also showed good ionic conductivity (Figure S14, Supporting Information), and a light‐emitting diode (LED) lamp worked regularly when the polymer electrolyte was used (Figure S15, Supporting Information).

## 3 Conclusion

In summary, we introduced EC–PC into the holographic photopolymer system and obtained a new polymer electrolyte with ordered ion channels. The loading of EC–PC was found to be capable of simultaneously increasing the diffraction efficiency and ionic conductivity. When loading 50 wt% of EC–PC in the system, a diffraction efficiency of 59.2 ± 11.7% and an ionic conductivity of 2.13 × 10^−4^ S cm^−1^ at 30 °C were afforded. They increased almost 60 times and 5 orders of magnitude in comparison with the pristine without EC–PC. Moreover, the introduction of DMAA increases the anisotropy of polymer holographic electrolytes. The formed holographic photopolymer electrolytes exhibited good toughness when bending and excellent transparency under ambient conditions. The solid‐state lithium‐ion batteries based on our holographic photopolymer electrolytes as the separator exhibited stable cycle performance. Finally, computer‐generated images were reconstructed in these new electrolytes. These findings pave a way to develop new flexible and transparent electronics with good mechanical, electrical, and optical functions.

## 4 Experimental Section

*Materials*: EC (98%, Adamas), PC (99%, Aldrich), DMAA (99%, Aldrich), PEGDA (*M*_n_ = 400, Aldrich), RB (85%, Acros), and NPG (97%, Aldrich) were used as received. Hyperbranched monomer in the brand of 6361‐100 was donated by Eternal Chemical Co., Ltd., China. LiClO_4_ (Aladdin) was dried in vacuum oven for 24 h at 120 °C before use. LiFePO and acetylene black (AB) were purchased locally. All other solvents were distilled under reduced pressure before use.

*Preparation of the Mixtures for Holography*: The mixtures for holography were required to be homogeneous and in low viscosity. EC‐PC (a complex liquid comprised of EC and PC), RB, NPG, PEGDA, 6361‐100, DMAA, and LiClO_4_ were added into a brown vial with a sealed cap, and then stirred for 5 h at room temperature. Subsequently, they were sonicated at 30 °C for 30 min to offer homogeneous mixtures. The employment of EC–PC was critical to completely dissolve the LiClO_4_, RB, and NPG. In addition, the EC–PC was able to maintain a low mixture viscosity, and the introduction of DMAA provided better fluidity for the precursor solution. Tables S1–S4 (Supporting Information) show the compositions for the holographic mixture with different EC–PC contents.

*Holographic Recording*: Photopolymer electrolytes with ordered ion channels were formed through holographic photopolymerization–induced phase separation upon exposure to laser interference field (Figure S16a, Supporting Information). Homogeneous mixtures were introduced into a glass cell with a cell gap of 100 µm. The cell was then vertically mounted on a sample holder. A 532 nm laser beam (Coherent, USA) was divided by a splitter into two beams with an equal intensity. These two beams were expanded and well‐collimated, then they simultaneously irradiated the samples from the same side to offer unslanted and uniform gratings with a pitch (*Λ*) of ≈740 nm (Figure S16b, Supporting Information). The intensity for each beam was 3.0 mW cm^−2^ and the recording time was 400 s. After holographic recording, a mercury lamp was employed to postcure the holographic photopolymer for 10 min.

*Characterization of Holographic Gratings*: The manufactured holographic photopolymer electrolyte was mounted on the sample holder of an LCT‐5016C display parameter tester, and probed by a nondestructive 633 nm He–Ne laser (Thorlabs, USA) from the Bragg angle (Scheme S1c, Supporting Information). The diffraction efficiency (η) was determined to be the ratio of diffraction intensity (*I*_d_) to the sum of diffraction and transmission (*I*_t_) intensities

(1) $$\eta =\frac{I_{d}}{I_{d}\;+\;I_{t}}$$

The Klein–Cook parameter (*Q*) of the holographic photopolymer electrolyte was calculated to be ≈176.1 (>10), indicative of volume holograms^20^

(2) $$Q=\frac{2\pi d\lambda_{\mathrm{writing}}}{n_{\mathrm{eff}}\Lambda^{2}}$$

where *d* was the photopolymer electrolyte thickness (100 µm), λ_writing_ was the writing wavelength (532 nm), and *n*_eff_ was the electrolyte average refractive index (≈1.45).

Then, the refractive index modulation *n* was calculated according to the Kogelnik's coupled wave theory^21^

(3) $$n=\frac{\arcsin\left(\eta^{0.5}\right)\lambda_{\mathrm{reading}}\cos\left(\theta_{B}\right)}{\pi d}$$

where λ_reading_ was the reading beam wavelength (633 nm) and θ_B_ was the Bragg angle.

*Differential Scanning Calorimetry*: The glass transition temperature (*T*_g_) of the holographic photopolymer electrolyte was measured by differential scanning calorimetry (DSC, Q2000, TA instruments). The holographic polymer electrolytes were cut into small specimens, then immersed in acetonitrile for 72 h to remove the EC and PC, and finally dried under vacuum at 80 °C. DSC data were obtained between −60 and 100 °C. About 8 mg specimens were added in one aluminum pan, then isothermally kept at −60 °C, finally heated to 100 °C at a ramp rate of 10 °C min^−1^ under the protection of nitrogen gas. Another empty aluminum pan was used as the reference. The photopolymerization kinetics of the holographic polymer monomer mixture was determined by photo‐differential scanning calorimeter (P‐DSC) (Q2000, TA instruments) with a monochromatic filter (full width at half maxima (FWHM) = 10 ± 2 nm, Andover Co., USA) at 532 nm illumination, and the light intensity was about 0.26 mW cm^−2^. About 10 g of the mixed liquid was dropped into the DSC aluminum pan by a pipette and placed on the DSC sample stage, under the N_2_ protection. It was kept at 30 °C for 5 min, and then exposed for 50 min. Another empty aluminum pan was used as the reference. The photopolymerization of the acrylic monomer/DMAA mixture was a process in which the C=C double bond was converted into a polymer chain, and the P‐DSC recorded the exothermic heat flow of this process. The photopolymerization rate *R*_P_ and the monomer conversion rate α were calculated according to the following formula^22^

(4) $$R_{P}=\left(\frac{dH}{dt}\right)/\left(\sum \frac{x_{i}wf_{i}\Delta H_{o}}{M_{i}}\right)$$

(5) $$\alpha =\Delta H/\left(\sum \frac{x_{i}wf_{i}\Delta H_{o}}{M_{i}}\right)$$

where d*H*/d*t* was exothermic heat flow, *w*_ _was the mass of acrylic monomer/DMAA, *x_i_*, *f_i_*, and *M_i_* were the mass fraction of monomers, number of functional groups, and molar mass, respectively, Δ*H* was the cumulative reaction heat flow up to the time (*t*), and Δ*H*_o_ was the standard reaction enthalpy of the C=C.

*Micromorphology Characterization*: The micromorphology of holographic photopolymer electrolytes was characterized by SEM (Sirion 200, FEI) and AFM (Shimadzu SPM‐9700). Holographic polymer electrolytes were cut into small specimens, and then soaked in acetonitrile for 72 h to completely remove the EC and PC, finally dried at room temperature. Prior to SEM characterization, the prepared samples were sprayed with a thin layer of platinum on the top surface. AFM analysis was conducted using an AFM setup on the bare surface. The tapping mode at a frequency of 300 kHz was employed during AFM characterization.

*Tensile Property Test*: The measurement was performed in the chamber with constant temperature and humidity on a such Tech UTM2103 universal testing machine with a 50 N loading cell at a strain rate 0.5 mm min^‐1^. The samples were tested with uniform specifications (12.5 mm × 0.1 mm).

*Electrochemical Impedance Spectroscopy*: The ionic conductivity (σ) of electrolyte membranes was measured by means of EIS on an electrochemical test system (MULTI AUTOLAB M204). The holographic photopolymer electrolyte was cut into a round‐shaped disk and sandwiched between two stainless steel electrodes. The EIS was measured with the sinusoidal amplitude modulation of 10 mV in the frequency range from 1 MHz to 100 Hz. The anisotropic conductivity of HPE was measured by adding copper electrodes on both sides of the film. Data were collected from 20 to 60 °C at an interval of 10 °C. Samples were held at each temperature for more than 30 min prior to measurement so that an equilibrium could be reached. The ionic conductivity values can be calculated by using the following equation

(6) $$\sigma =\frac{l}{R_{b}S}$$

where *l* was the thickness of the electrolyte film, S was the cross‐sectional area between the electrolyte and stainless‐steel electrode, and *R*_b_ was the bulk resistance of the electrolyte that was obtained from the EIS.

*Cell Assembly and Electrochemical Measurements*: The symmetric Li/HPE/Li coin cells (2032 type) were evaluated by galvanostatic cycling experiments at 60 °C to study the lithium electrodeposition. The electrochemical stability of the holographic polymer electrolytes was determined via LSV of the Li/HPE/SS cells at a 1 mV s^−1^ of scan rate over the 0–7 V range at 60 °C. To characterize the battery performance based on the holographic photopolymer electrolyte, LiFeO_4_ was employed as the active cathode material. The cathode material was composed of LiFePO_4_, acetylene black, and polyvinylidene fluoride (PVDF) in a weight ratio of 8:1:1. The holographic photopolymer electrolyte and cathode material were cut into a round‐shape disk (diameter: 16 mm) for assembling the coin‐cell full batteries (2032 type). Metal lithium was used as the anode. The charge/discharge performance was characterized under the voltage range of 2.5–4.2 V on the LAND CT2001A Battery Cycler (Wuhan, China).

*Image Storage in Holographic Photopolymer Electrolyte*: Different from the optical setup to record uniform holographic gratings (Figure S16b, Supporting Information), during the holographic image reconstruction, a spatial light modulator and a lens were used to project the computer‐generated holographic images into space as the object beam (Figure S16d, Supporting Information). Images were replicated in the photopolymer electrolyte by interference of the object and reference beams.

## Conflict of Interest

The authors declare no conflict of interest.

## Supporting information

Supplementary
