# Supplementary material for: Monochromatic “Photoinitibitor”‐Mediated Holographic Photopolymer Electrolytes for Lithium‐Ion Batteries
Source: Adv Sci (Weinh). 2019 Apr 4;6(10):1900205. doi: 10.1002/advs.201900205 (PMC6524123; doi:10.1002/advs.201900205)
Supplement: Supplementary file 1 — Supplementary [file ADVS-6-1900205-s001.pdf]

Copyright WILEY-VCH Verlag GmbH & Co. KGaA, 69469 Weinheim, Germany, 2019.

ADVANCED SCIENCE
Open Access

# Supporting Information

for *Adv. Sci.,* DOI: 10.1002/advs.201900205

# Monochromatic "Photoinitibitor"-Mediated Holographic Photopolymer Electrolytes for Lithium-Ion Batteries

*Ronghua Yu, Sibo Li, Guannan Chen, Cai Zuo, Binghua Zhou, Mingli Ni, Haiyan Peng,* Xiaolin Xie, and Zhigang Xue**

Copyright WILEY-VCH Verlag GmbH & Co. KGaA, 69469 Weinheim, Germany, 2019.

# Supporting Information

**Monochromatic “Photoinitibitor”-Mediated Holographic Photopolymer Electrolytes for Lithium-Ion Batteries**

*Ronghua Yu, Sibo Li, Guannan Chen, Cai Zuo, Binghua Zhou, Mingli Ni, Haiyan Peng,* Xiaolin Xie, and Zhigang Xue**

**Table S1.** Compositions of Holographic Mixtures.

| EC-PC [wt%] | PEGDA [wt%] | 6361-100 [wt%] | $LiClO_4$ [wt%] | RB [wt%] | NPG [wt%] |
|---|---|---|---|---|---|
| 0 | 66.7 | 33.3 | 9.0 | 0.6 | 1.3 |
| 10.0 | 60.0 | 30.0 | 9.0 | 0.6 | 1.3 |
| 20.0 | 53.3 | 26.7 | 9.0 | 0.6 | 1.3 |
| 30.0 | 46.7 | 23.3 | 9.0 | 0.6 | 1.3 |
| 40.0 | 40.0 | 20.0 | 9.0 | 0.6 | 1.3 |
| 50.0 | 33.3 | 16.7 | 9.0 | 0.6 | 1.3 |

**Table S2.** Ionic Conductivity at 30 and 80 °C, and Fitted Parameters for Arrhenius Equation of Holographic Photopolymer Electrolyte (HPE) with Varied Contents of EC-PC.

| EC-PC [wt%] | σ at 30 °C [S cm$^{-1}$] | σ at 80 °C [S cm$^{-1}$] | $E_a$ [KJ mol$^{-1}$] | σ [S cm$^{-1}$] |
|---|---|---|---|---|
| 0 | $2.01\times10^{-9}$ | $9.32\times10^{-7}$ | 109.03 | $1.70\times10^{10}$ |
| 10.0 | $1.45\times10^{-8}$ | $1.81\times10^{-6}$ | 85.80 | $1.06\times10^{7}$ |
| 20.0 | $3.93\times10^{-7}$ | $2.08\times10^{-5}$ | 68.93 | $3.38\times10^{5}$ |
| 30.0 | $3.24\times10^{-6}$ | $5.02\times10^{-5}$ | 48.71 | 912.01 |
| 40.0 | $1.59\times10^{-5}$ | $1.86\times10^{-4}$ | 42.63 | 354.81 |
| 50.0 | $2.13\times10^{-4}$ | $1.10\times10^{-3}$ | 27.48 | 12.11 |

**Table S3.** Compositions of Holographic Mixtures with Different Ratios of EC to PC.

| EC [wt%] | PC [wt%] | PEGDA [wt%] | 6361-100 [wt%] | $LiClO_4$ [wt%] | RB [wt%] | NPG [wt%] |
|---|---|---|---|---|---|---|
| 0 | 50.0 | 33.3 | 16.7 | 9.0 | 0.6 | 1.3 |
| 10.0 | 40.0 | 33.3 | 16.7 | 9.0 | 0.6 | 1.3 |
| 20.0 | 30.0 | 33.3 | 16.7 | 9.0 | 0.6 | 1.3 |
| 25.0 | 25.0 | 33.3 | 16.7 | 9.0 | 0.6 | 1.3 |
| 30.0 | 20.0 | 33.3 | 16.7 | 9.0 | 0.6 | 1.3 |

| | | | | | | |
|---|---|---|---|---|---|---|
| 40.0 | 10.0 | 33.3 | 16.7 | 9.0 | 0.6 | 1.3 |

**Table S4.** Compositions of Holographic Mixtures with various DMAA Contents.

| EC-PC [g] | PEGDA [g] | 6361-100 [g] | DMAA [g] | $LiClO_4$ [g] | RB [g] | NPG [g] |
|---|---|---|---|---|---|---|
| 0.6 | 0.333 | 0.167 | 0 | 0.09 | 0.006 | 0.013 |
| 0.6 | 0.333 | 0.167 | 0.1 | 0.108 | 0.0066 | 0.0143 |
| 0.6 | 0.333 | 0.167 | 0.2 | 0.126 | 0.0084 | 0.0182 |

**Table S5.** Bragg Angle ($\theta_B$), Diffraction Efficiency ($\eta$), and Refractive Index Modulation (n) of HPE with Varied Content of EC-PC.

| EC-PC [wt%] | $\theta_B$ [°] | $\eta$ [%] | n |
|---|---|---|---|
| 0 | - | 0 | - |
| 10.0 | 20.0±0.8 | 0.9±0.9 | 0.00018 |
| 20.0 | 19.9±0.4 | 5.0±0.9 | 0.00043 |
| 30.0 | 20.1±0.5 | 23.1±8.3 | 0.00095 |
| 40.0 | 20.1±0.5 | 42.4±3.2 | 0.00134 |
| 50.0 | 19.5±0.7 | 59.2±11.7 | 0.00167 |
| $DMAA_{0.1}$ | 19.6±0.8 | 73.5±10.4 | 0.00195 |
| $DMAA_{0.2}$ | 19.7±0.8 | 53.9±14.8 | 0.00156 |

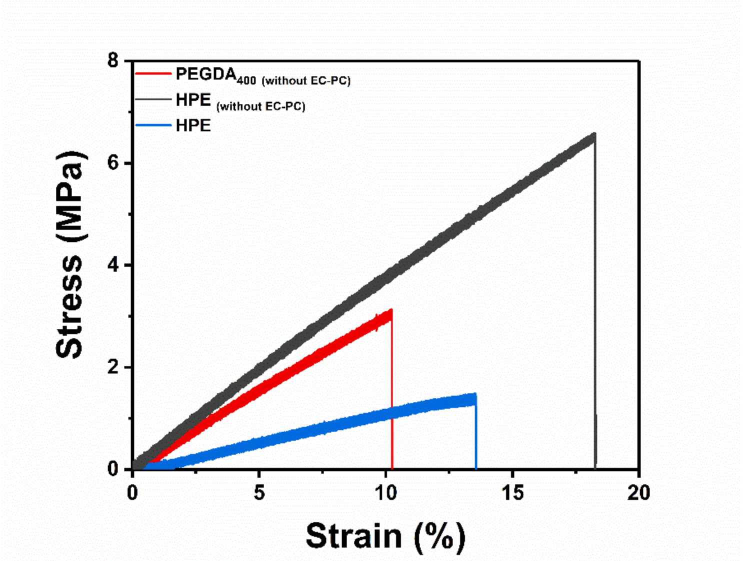


**Figure S1.** Stress–strain curves of HPE (without EC-PC), HPE (with 50 wt % EC-PC) and $PEGDA_{400}$ (after crosslink).

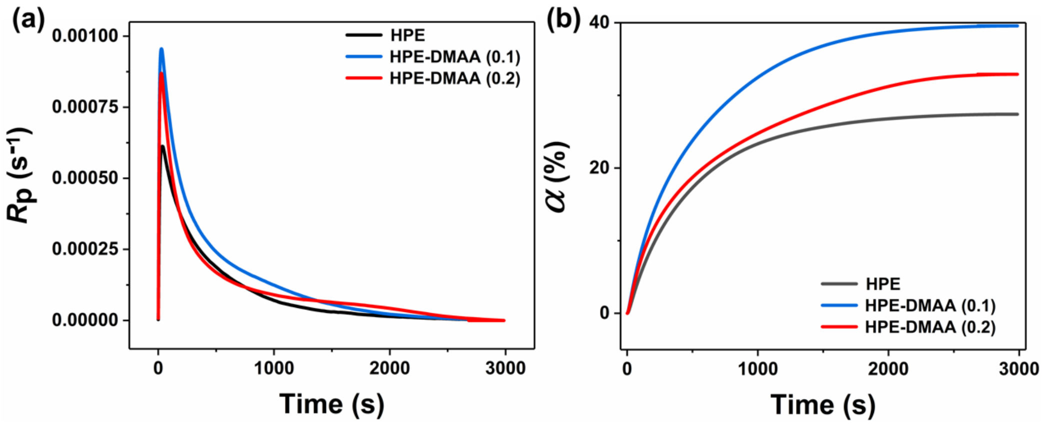


**Figure S2.** (a) Photopolymerization rate of monomer mixture at a wavelength of 422 nm with the light intensity of 0.26 mW/cm$^2$; (b) the relationship between monomer conversion rate and reflection time.

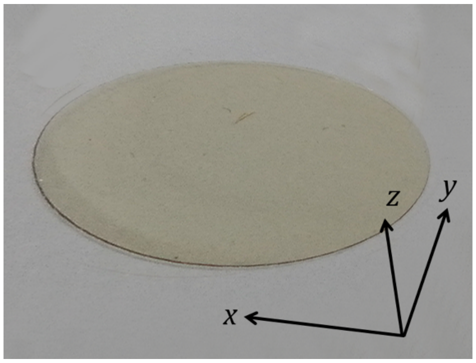


**Figure S3.** Schematic of the *xy* plane (parallel to the film surface) and the *xz* plane (vertical to the film surface) of the films.

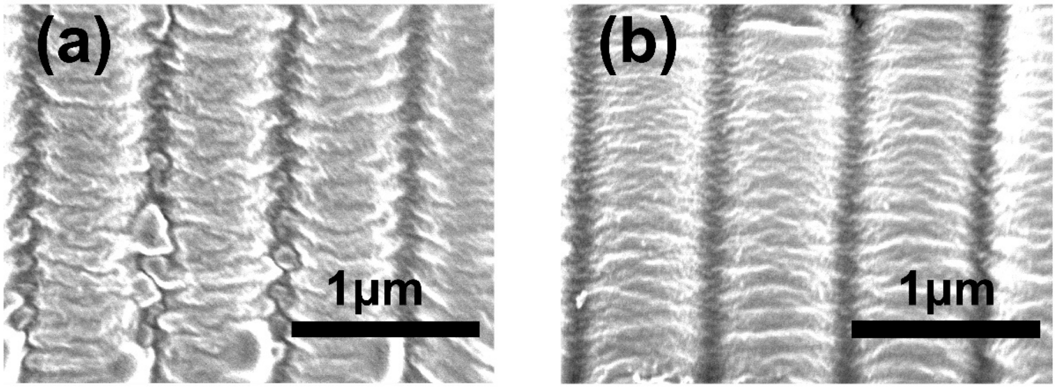


**Figure S4.** SEM images of the HPE：(a) anisotropic electrolytes with 50 wt% of EC-PC, (b) anisotropic electrolytes with 50 wt% of EC-PC and 0.1 g of DMAA.

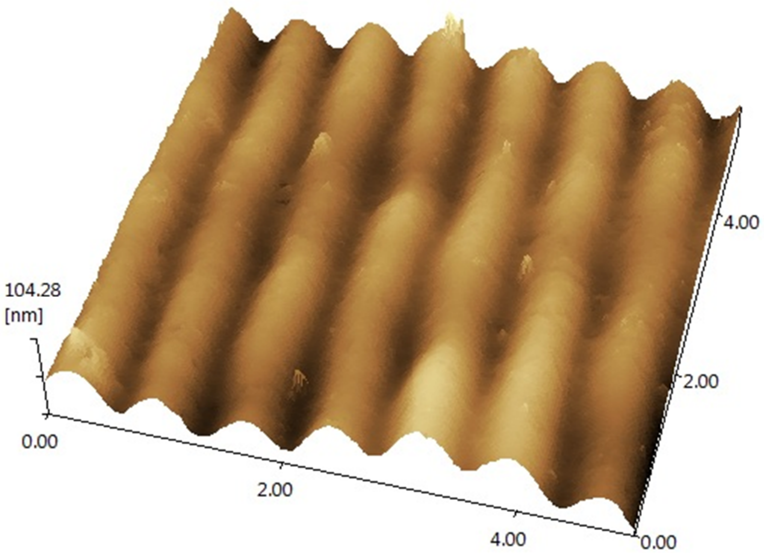


**Figure S5.** AFM image of anisotropic electrolytes with 50 wt% of EC-PC and 0.1 g of DMAA.

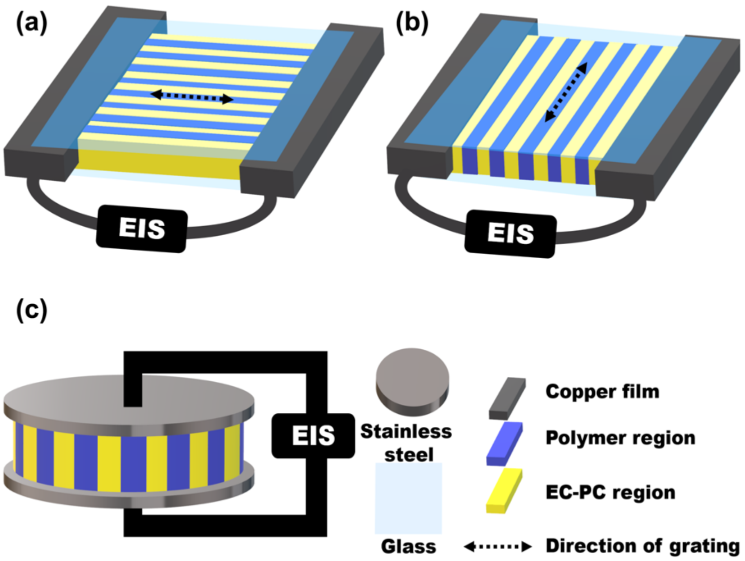


**Figure S6.** Schematic illustration of conductivity testing for HPE with ordered structures in different directions. (a) parallel to the grating (||); (b) vertical to the grating (⊥); (c) HPE film for ionic conductivity testing.

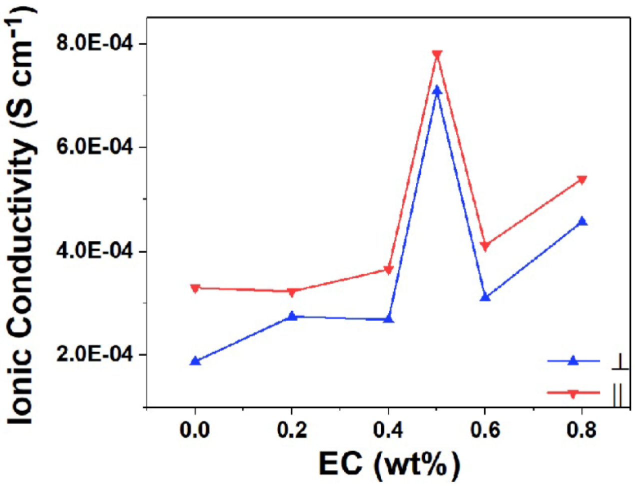


**Figure S7.** Anisotropic conductivity of HPE with varied content of EC to PC, at 20 $^{o}$C.

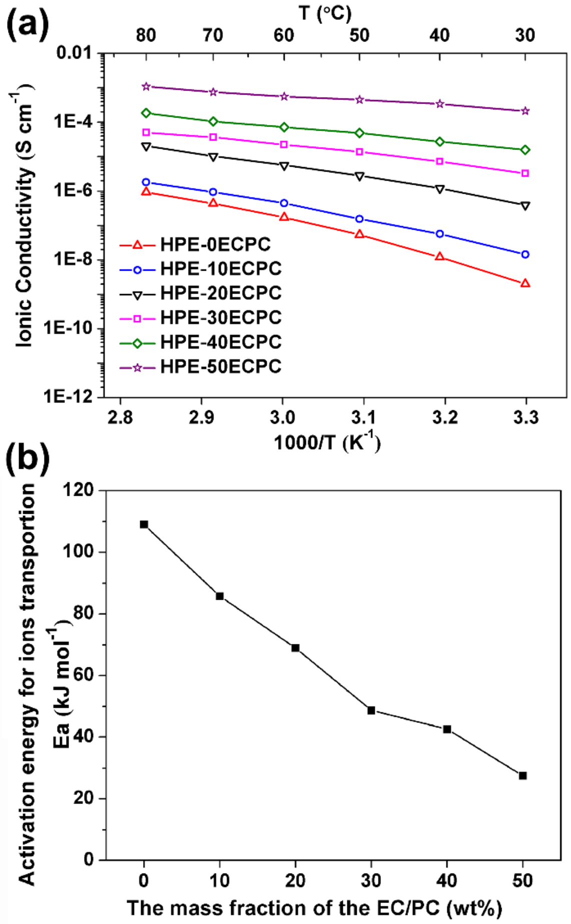


**Figure S8.** (a) Temperature dependence of ionic conductivity for HPEs with various mass fraction of EC-PC. (b) The activation energy for HPEs with various mass fraction of EC-PC.

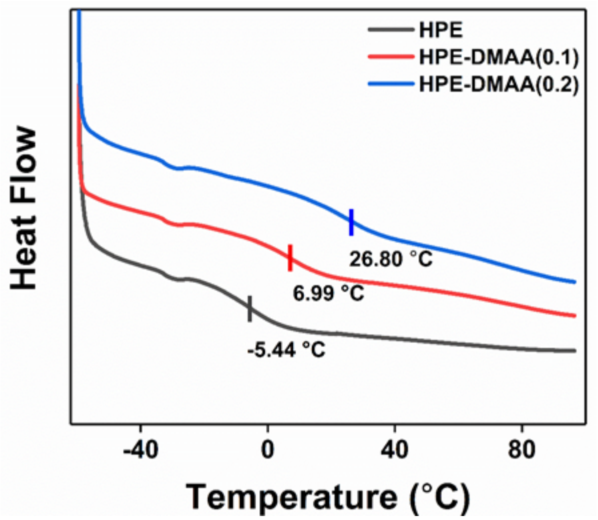


**Figure S9.** DSC curves of HPE and HPE-DMAA.

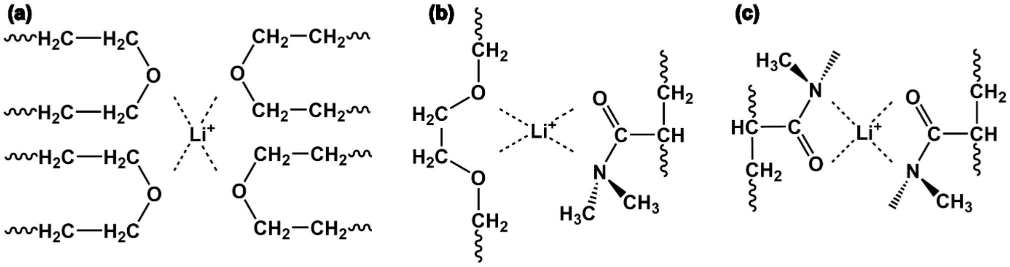


**Figure S10.** Schematic structure of the complexes formed by $Li^+$ cation with (a) ethoxylated segments (type I complexes), (b) ethoxylated segments and acrylamide chains (type II complexes), and (c) acrylamide chains (type III complexes).

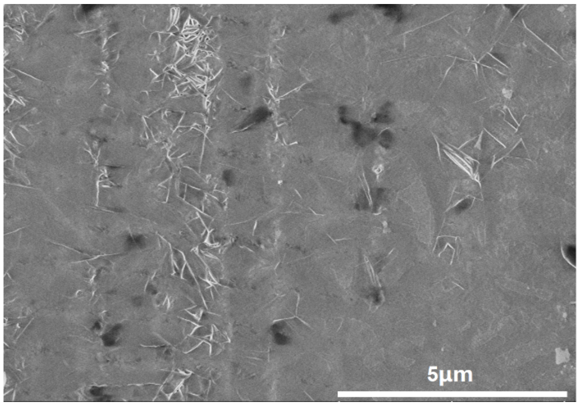


**Figure S11.** SEM image of Li mental surface at a certain current density of 0.05 mA $cm^{-2}$ after 2000 h.

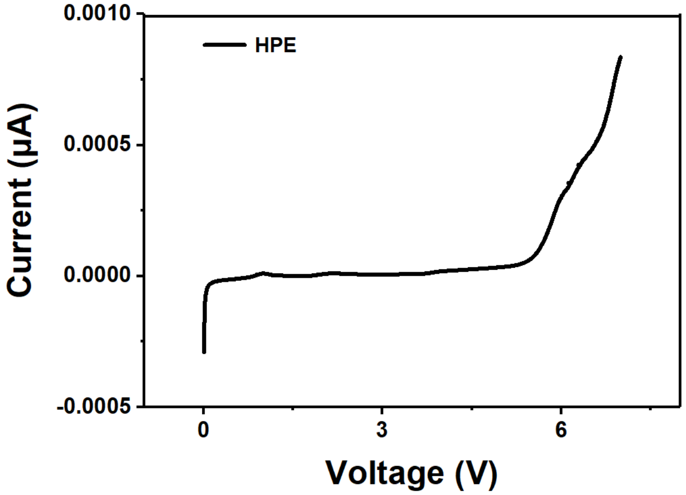


**Figure S12.** Linear sweep voltammograms of HPE on a working electrode of stainless-steel and a counter and reference electrode of lithium metal at a scan rate of 0.1 mV $s^{-1}$.

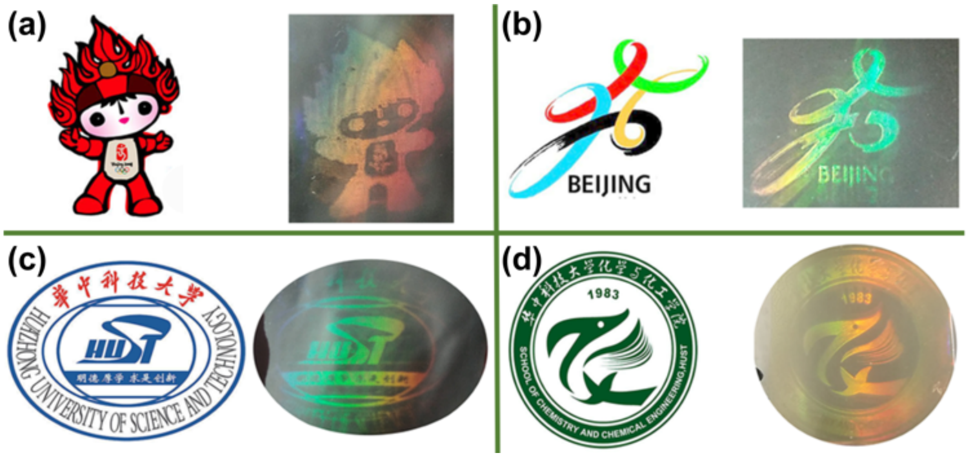


**Figure S13.** Original computer-generated images and replicated images in the HPE. (a) Fuwa Huanhuan (official mascot of Beijing 2008 Olympic Games), (b) emblem of Beijing 2008 Olympic Bid Committee, (c) logo of HUST, (d) logo of School of Chemistry and Chemical Engineering of HUST.

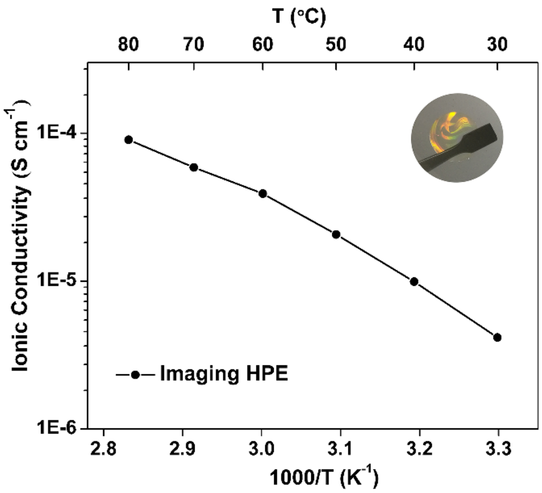


**Figure S14.** (a) Temperature dependence of ionic conductivity for HPEs with an image storage in the case of 30 wt% EC-PC.

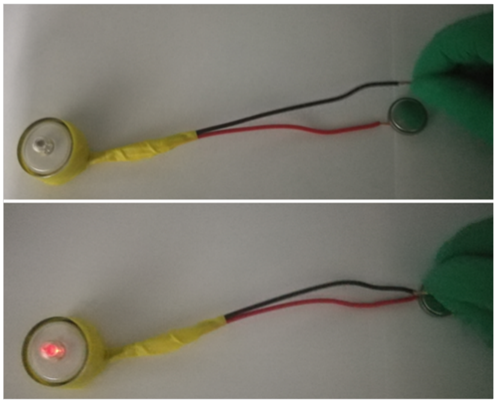

**Figure S15.** Photographs to show the cell lighting up an LED lamp.

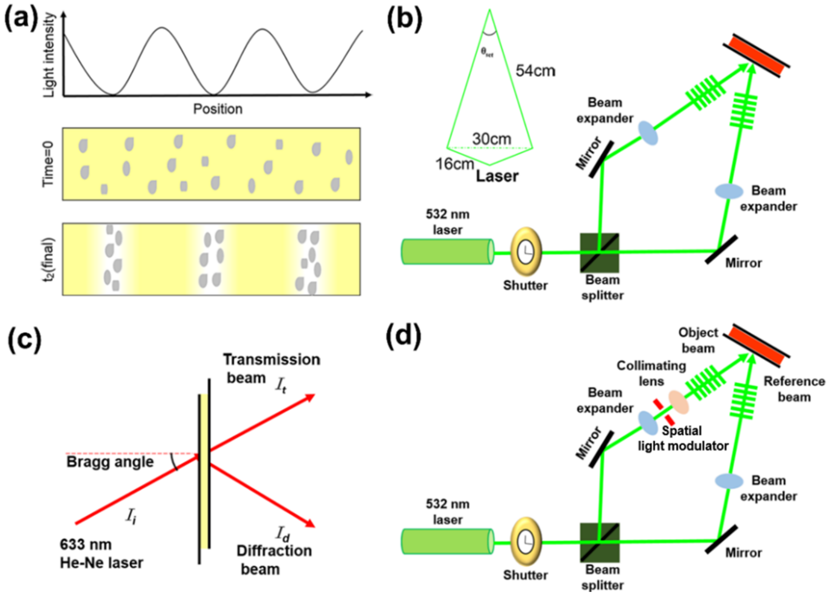


**Figure S16.** Schematic Illustration on Holographic Recording. (a) Holographic photopolymerization-induced phase separation upon sinusoidal interference light patterns. (b) Optical setup for forming uniform holographic photopolymer electrolyte. (c) Diffraction efficiency characterization of the holographic polymer electrolyte at the Bragg angle using a nondestructive 633 nm laser. (d) Holographic image storage in the photopolymer electrolyte.
